# Gene co-expression network construction and analysis for identification of genetic biomarkers associated with glioblastoma multiforme using topological findings

**DOI:** 10.1186/s43046-023-00181-4

**Published:** 2023-07-24

**Authors:** Seema Sandeep Redekar, Satishkumar L. Varma, Atanu Bhattacharjee

**Affiliations:** 1 Pillai College of Engineering, New Panvel, Mumbai, India; 2 SIES Graduate School of Technology, Navi Mumbai, Mumbai, India; 3 Pillai College of Engineering, New Panvel, Mumbai, India; 4 Leicester Real World Evidence Unit, University of Leicester, Leicester, UK

**Keywords:** Cancer progression, Genetic biomarker, Survival, Correlation, Network topology, Graph theory

## Abstract

**Background:**

Glioblastoma multiforme (GBM) is one of the most malignant types of central nervous system tumors. GBM patients usually have a poor prognosis. Identification of genes associated with the progression of the disease is essential to explain the mechanisms or improve the prognosis of GBM by catering to targeted therapy. It is crucial to develop a methodology for constructing a biological network and analyze it to identify potential biomarkers associated with disease progression.

**Methods:**

Gene expression datasets are obtained from TCGA data repository to carry out this study. A survival analysis is performed to identify survival associated genes of GBM patient. A gene co-expression network is constructed based on Pearson correlation between the gene’s expressions. Various topological measures along with set operations from graph theory are applied to identify most influential genes linked with the progression of the GBM.

**Results:**

Ten key genes are identified as a potential biomarkers associated with GBM based on centrality measures applied to the disease network. These genes are SEMA3B, APS, SLC44A2, MARK2, PITPNM2, SFRP1, PRLH, DIP2C, CTSZ, and KRTAP4.2. Higher expression values of two genes, SLC44A2 and KRTAP4.2 are found to be associated with progression and lower expression values of seven gens SEMA3B, APS, MARK2, PITPNM2, SFRP1, PRLH, DIP2C, and CTSZ are linked with the progression of the GBM.

**Conclusions:**

The proposed methodology employing a network topological approach to identify genetic biomarkers associated with cancer.

## Introduction

Among all different types of cancer, GBM is one of the most common brain tumors with high mortality. It is the most aggressive primary intracranial tumor, displaying heterogeneity and rapid proliferation [1]. There are various treatment options available to treat GBM such as surgical resection, chemotherapy, and radiotherapy; it is still a deadly disease with a rapid prognosis. Patients usually have a median survival of approximately 14 to 15 months from the date of diagnosis [2]. Overall survival of the GBM patients is also very poor. Therefore, developing an appropriate and effective strategy to analyze the overall survival of the GBM patient and determine the progression of the disease is very crucial.

Tumor development is a complex pathological process that involves multiple genetic alterations [3]. In most cases of cancer, these alterations happen one after another in different genes in a specific group of cells over time to cause malignancy. That is the reason genes are considered to be one of the causes behind cancer development and progression. Therefore, understanding more about these genetic markers is essential in cancer study. It helped exceptionally to improve the early diagnosis and prognosis of the disease [4, 5].

Deciphering the biological networks underlying cancer is undoubtedly important for understanding the molecular mechanisms of the disease and identifying effective biomarkers to determine cancer progression [6]. Achievements in identifying potential biomarkers by constructing gene co-expression networks encourage researchers to study the direct possible relationship between genes and disease.

GBM datasets from the TCGA (The Cancer Genome Atlas) repository that include a large number of molecular features [7] is obtained for this study. A genomic dataset known as the DNA methylation dataset is obtained from TCGA which is preprocessed and prepared for the survival analysis [8]. Survival-associated genes are identified through time to event data analysis which is considered for further analysis. A separate gene co-expression network is constructed from 156 survival outcome associated genes identified. For constructing the biological network, three different correlation measures are used; those are ‘Pearson’, ‘Spearman,’ and ‘Kendall’ [9]. Structural and topological properties of the network are useful to analyze these networks because network topologies describe the ways in which the elements of a network are connected as well as how they communicate. Network centrality measures are found useful in network analysis [10]. These are used to determine the importance of the node in the network; it signifies a type of flow or transfer across the network. This allows centralities to be classified by the type of flow they consider important [11]. Centrality can also be interpreted as involvement in the cohesiveness of the network. This allows centralities to be classified based on how they measure cohesiveness [12]. Various centrality measures are found useful in network analysis; those are degree centrality, eigenvector centrality, closeness centrality, and betweenness centrality to identify biomarkers that are closely associated with the progression of glioma [13]. Degree centrality of a node determines how many other nodes in the network it is connected with. Betweenness centrality of a node is the sum of the fraction of all-pairs shortest paths that pass through the node. Closeness centrality measures how short the shortest paths are from node i to all the other nodes in the network. Eigenvector centrality of a particular node is based on the centrality of its neighbors. There are other centrality measures available in the literature. In order to perform an analysis of gene co-expression networks, these 4 are found to be more relevant and important [14, 15].

## Materials and methods

### Data collection and preprocessing

GBM datasets were downloaded from TCGA (https://portal.gdc.cancer.gov/). TCGA repository is a rich source of multiple omics data represented by varied genomic profiles. The DNA methylation profile from human glioblastoma samples was obtained from the GDC portal. TCGA DNA methylation data of GBM patients comes with epigenetic markers that are helpful to understand suspected regulatory roles in disease progression [16]. The GBM datasets obtained from TCGA required filtering out lower quality data points and outliers [7]. It is also necessary to perform pre-processing steps like cleaning to remove data inconsistency, data transformations, and data reduction as per the requirement of the study to conduct survival analysis [17].

### Methodology

In the first step of implementation, Preprocessing is performed on the GBM dataset which consists of a gene identifier, expression value for each gene for 76 GBM patients along with their survival information. Genomic data get produced at the rate of 10 terabytes a day and require complicated processing to transform massive amounts of noisy raw data into biological information [18]. It is very essential to perform end-to-end processing of genomic data, which includes data aligning, variation discovery, and deep analysis. In this study also, filtering is integrated into the preprocessing phase in order to prepare data for applying appropriate techniques for identifying survival outcome associated genes [19].

**Fig. 1:**
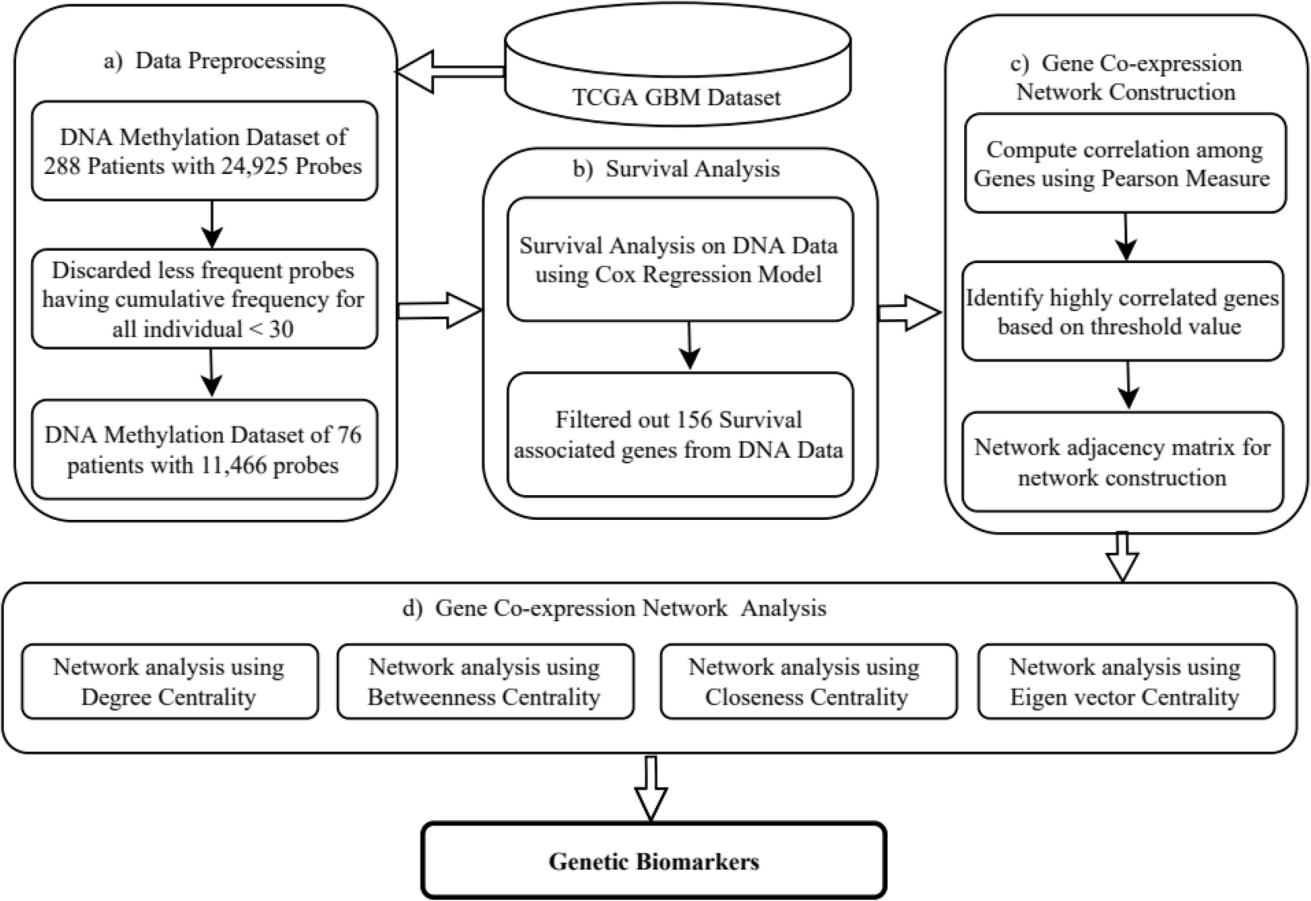
Workflow of the methodology used for identification of genetic biomarker. **a** Data pre-processing steps. **b** Survival analysis. **c** Gene co-expression network construction steps. **d** Gene co-expression network analysis

A method known as survival analysis is implemented to identify genes associated with the cancer patient’s overall survival. Various methods of non-parametric, semi-parametric, and parametric methods of survival analysis are studied and analyzed to find one which is to be applied finally. The Cox proportional hazards regression model is used to identify possible factors associated with patients’ overall survival [20]. However, overall survival (OS) is defined as time between dates of diagnosis till date of death or last follow up. In entire study we assume that progression of the disease is represented by earlier death, so patient died earlier just because rapid progression of the disease corresponding to the penetrating genes (genetic biomarkers) associated for it. So, we explored the penetrating genes for death and defined as disease progressive genes. This model is used to evaluate the effect of those factors and subsequently examine how a genetic marker controls the rate of a particular event (e.g., death) at a specific point in time. This is termed as hazard rate. Influencing factors are covariates in the survival-analysis literature. Cox proportional hazards regression model is applied to the DNA methylation data. Out of 24,925 genes, 156 are identified as significant genes (*p* value ≤ 0.01) associated with the patient’s overall survival from the genomic dataset as an outcome of this step as shown in Fig. 1. Gene co-expression networks are constructed from these 156 genes extracted from the DNA Methylation dataset [21].

In the third step, the gene co-expression network is constructed by using three methods of correlation measure, i.e., Pearson, Kendall, and Spearman. Correlation measures are selected in order to establish a link between two significant genes while constructing a network [22]. A threshold value is selected arbitrarily to focus on moderate correlation to represent in the network. Finally Pearson correlation is used for network construction. In the fourth step, the constructed gene co-expression network is analyzed using structural and topological properties of the network such as degree centrality, closeness centrality, betweenness centrality, and eigenvector centrality. A set theory is applied in which the operations such as intersection, union, and difference are performed for the identification of the most influential genes associated with GBM progression. The detail method is shown in Fig. 1.

### Construction of the gene co-expression network

**Fig. 2:**
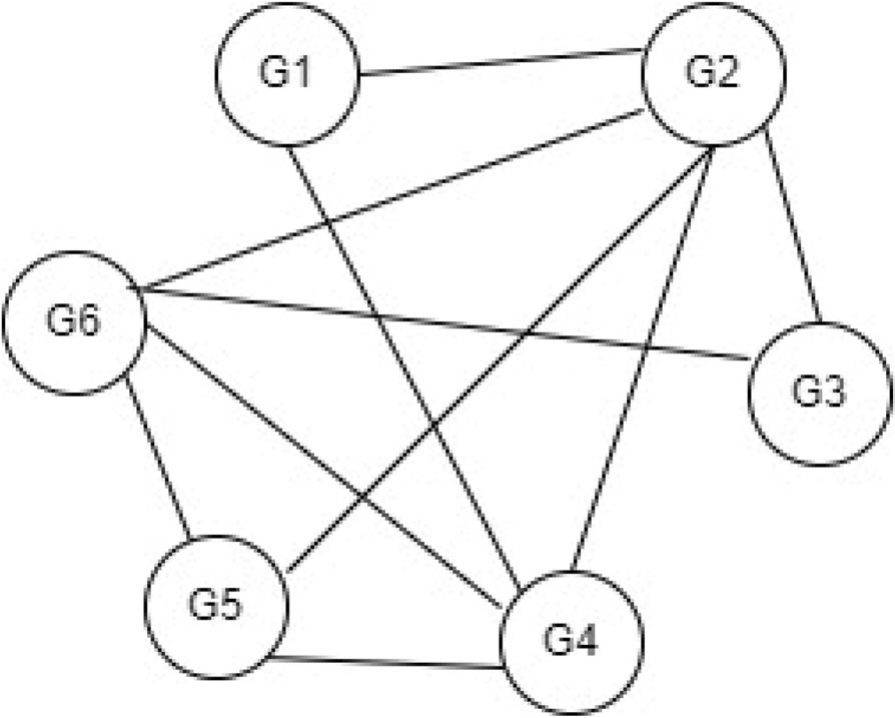
Gene co-expression network comprising 6 genes and connection between genes indicates that they have significant correlation

A set of co-expressed genes produces proteins. The correlation among the genes expressed in different biological conditions is captured by gene co-expression networks [23]. It is represented as an undirected graph *G* = (*V*, *E*) where the *V* represents genes, *E* represents the edge connecting two genes that are significantly co-expressed. An edge connecting a pair of nodes indicates that the corresponding genes have significantly similar expression patterns, which in turn indicate that genes are active under the same biological condition. The gene co-expression network is shown in Fig. 2 which consists of 6 genes (G1, G2, …, G6). The connection between genes shows that they have similar expression patterns which mean that they have a high correlation (above the threshold). Co-expressed genes are very important from the biological point of view as they are controlled by the same transcriptional regulatory program, they show functional relations or they are members of the same pathway or protein complex.

**Fig. 3:**
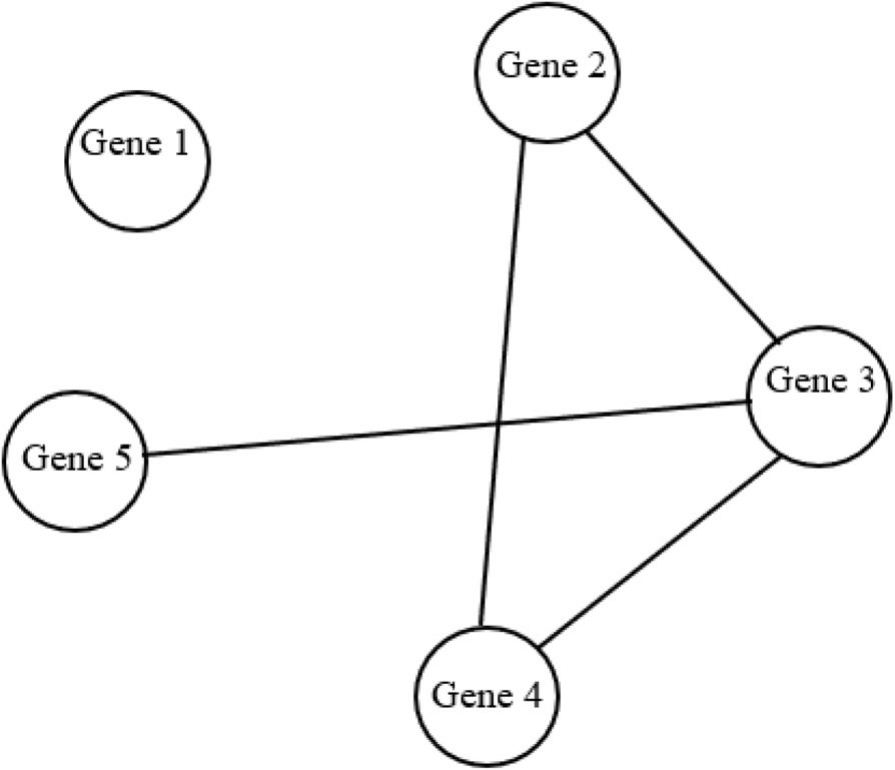
Gene co-expression network based on adjacency matrix shown in Table 1(c)

Many methods are developed for constructing gene co-expression networks [24, 25], which basically follow a two-step approach. In the first step, for every pair of genes, a similarity score is calculated using an appropriate co-expression measure. Then, a pair of genes is linked by an edge in the network having correlation scores more than the selected threshold which shows that the gene pair has a significant co-expression relationship. For developing gene co-expression network, matrix form is used to provide input. The *m* × *n* matrix represents genes and n samples. A stepwise process of gene co-expression network construction is shown in Table 1. We have shown this process on top five genes out of 156 genes ( having *p* value < 0.01) obtained through survival analysis in order to simplify the network construction process. The same set of steps is followed for gene co-expression network construction with 156 genes. Step 1(a) shows survival outcome associated genes identified through cox regression analysis with their expression values. Correlation value (up to two decimal points) between every gene is presented in step 1(b) which is calculated using Pearson correlation measure. Network adjacency matrix is obtained as shown in step 1(c), based on arbitrary threshold value as 0.5. If the correlation value is above 0.5, it is indicated by ‘1’ otherwise it is ‘0’. Figure 3 shows constructed gene co-expression network based on adjacency matrix of step 1(c). Value “1” in the adjacency matrix represents link (correlation) between two nodes (genes) and “0” represents absence of link which indicates that there is no significant correlation exist between pair of genes. For better visualization, subset of dataset is selected (i.e., 25 genes out of 156 survival outcome associated genes) for representation gene co-expression network [26, 27].

**Table 1:** Gene co-expression network construction steps (a) five genes with their expression values (b) co-relation matrix showing correlation among five genes (c) network adjacency matrix where correlation above threshold (> 0.5) is presented as ‘1’, otherwise it is ‘0’

(a) Genes with the expression values	Sample ID\Gene ID	Gene expression values				
		cg25226014 (Gene 1)	cg00176210 (Gene 2)	cg04369341 (Gene 3)	cg00936626 (Gene 4)	cg01354473 (Gene 5)
	TCGA.02.0047.01A	0.85	0.82	0.70	0.82	0.84
	TCGA.02.0055.01A	0.57	0.69	0.17	0.89	0.56
	TCGA.02.2483.01A	0.91	0.87	0.83	0.86	0.84
	TCGA.02.2485.01A	0.91	0.90	0.41	0.90	0.75
	TCGA.02.2486.01A	0.84	0.64	0.65	0.49	0.70
(b) Co-relation matrix	Gene ID	Correlation values				
		cg25226014 (Gene 1)	cg00176210 (Gene 2)	cg04369341 (Gene 3)	cg00936626 (Gene 4)	cg01354473 (Gene 5)
	cg25226014 (Gene 1)	1.00	0.15	0.23	-0.01	0.06
	cg00176210 (Gene 2)	0.15	1.00	0.55	0.54	0.42
	cg04369341 (Gene 3)	0.23	0.55	1.00	0.50	0.51
	cg00936626 (Gene 4)	-0.01	0.54	0.50	1.00	0.30
	cg01354473 (Gene 5)	0.06	0.42	0.51	0.30	1.00
(c) Network adjacency matrix	Gene ID	Significant correlation indicated as ‘1’				
		cg25226014 (Gene 1)	cg00176210 (Gene 2)	cg04369341 (Gene 3)	cg00936626 (Gene 4)	cg01354473 (Gene 5)
	cg25226014 (Gene 1)	1	0	0	0	0
	cg00176210 (Gene 2)	0	1	1	1	0
	cg04369341 (Gene 3)	0	1	1	1	1
	cg00936626 (Gene 4)	0	1	1	1	0
	cg01354473 (Gene 5)	0	0	1	0	1

### Overall analysis

There are different network-based measures on the basis of which gene co-expression network can be analyzed [28]. Centrality measures are an important tool in social and complex network analysis to quantify the eminence of nodes. A centrality measure is an estimation of the structural importance of a node based on its location, connectivity, or any other structural property. Several measures are coined in literature. Among all, centrality measure is found to be important to identifying most influential nodes depicting biomarker genes associated with progression of the disease from disease network.

**Fig. 4:**
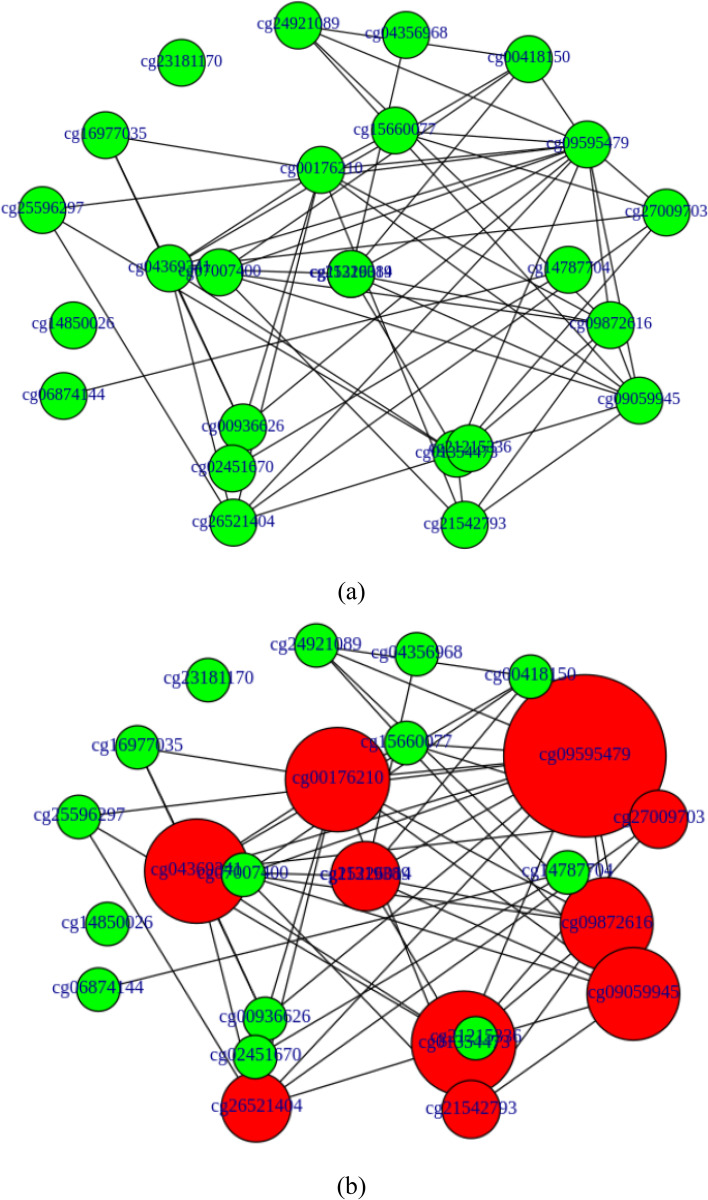
Gene co-expression network analysis using degree centrality. **a** Gene co-expression network. **b** Top 10 nodes with high degree centrality are shown with red color and size of those nodes varies as per the degree centrality value

Gene co-expression network constructed in the earlier step is analyzed using different centrality measures. For the simplicity we have shown the network by considering top 25 genes (*P*_value), out of 156 genes identified in the previous steps. Figure 4a shows the initial network of genes and their correlation computed through Pearson measure. Similarly the network is constructed using other two correlation measures which are Spearman and Kendall [29, 30]. There are various approaches used for analysis of gene co-expression network. Topological properties are found useful in network analysis. Among all other measures centrality measures are important to apply on the network to decide importance of the node within the network. In this study, three centrality measures namely degree centrality, closeness centrality, betweenness centrality, and eigenvector centrality are applied on the gene co-expression network to determine each node’s (gene’s) significance in the disease network [31]. The graph visualization method is used to show the nodes with higher degree centrality value in red color and in varied size according to their respective degree centrality value in Fig. 4b. Similarly other centrality measures are applied independently on the each gene co-expression network constructed using three different correlation criteria.

**Fig. 5:**
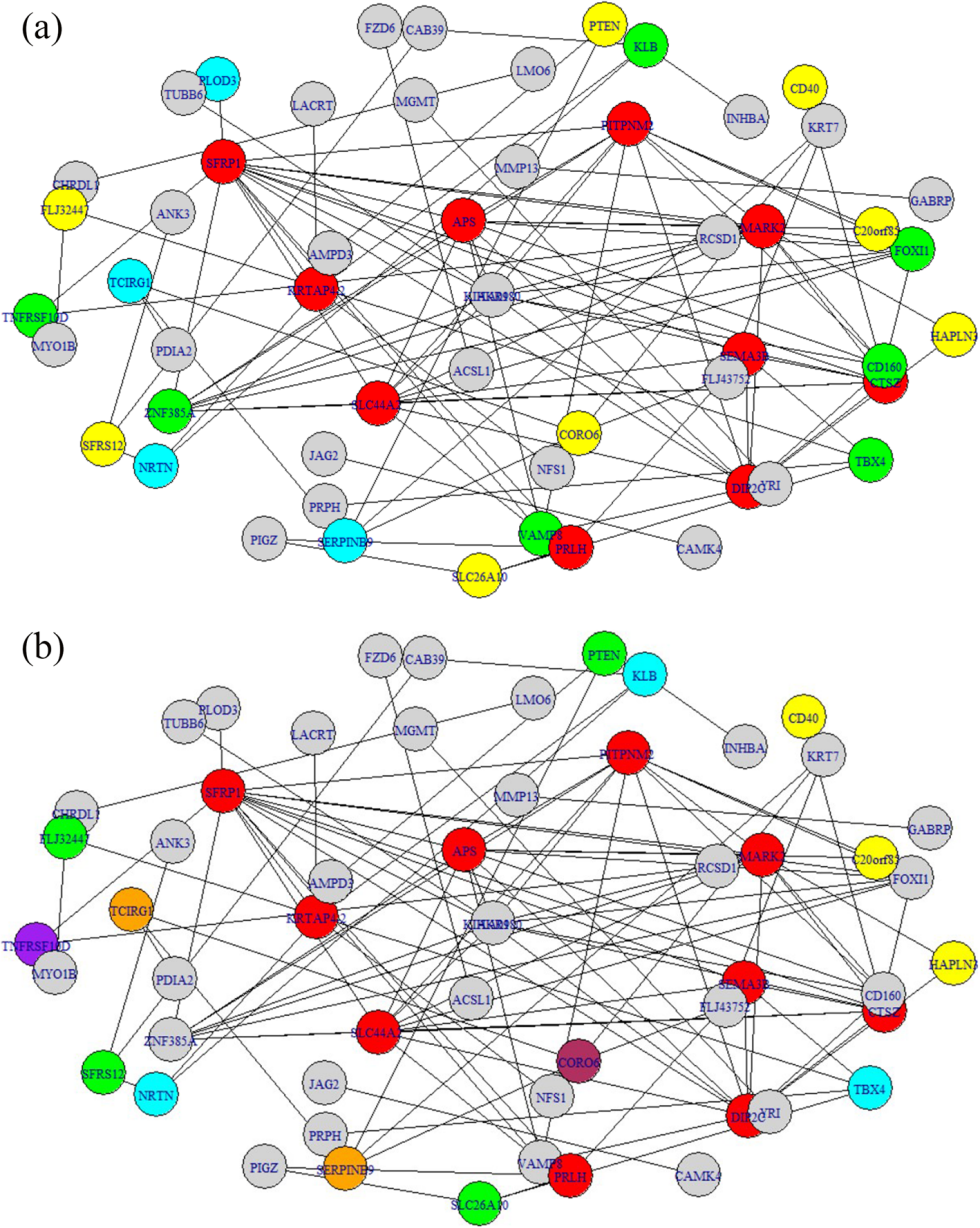
Gene co-expression network analysis using centrality measures, nodes shown in red color satisfies all four centrality criteria’s which signifies most influential genes. **a** Overall analysis. **b** Weighted analysis

In a similar way, the overall analysis is performed on the network constructed using three measures: Pearson, Spearman, and Kendall. In all the networks, networks constructed using the Pearson correlation measures are found to be more appropriate to analyze further. All four centrality measures are applied on this network and the set of nodes that are satisfying all the four centrality measures are considered to be the most significant which are shown in a red color node in Fig. 5a.

### Weighted analysis

Weighting is a statistical technique in which datasets are manipulated through calculations in order to bring them more in line with the population being studied [32]. It allows researchers to correct issues that occurred during data collection. For this reason, weighting is also known as post-stratification, as it takes place after the sample has been selected. It is referred to as statistical adjustments that are made in order to improve the accuracy of the survey estimates [33]. In this study, we have performed a weighted analysis so as to verify the accuracy of the overall analysis performed in the earlier step.

To decide the importance of the node in the gene co-expression network, we have applied four centrality measures which are degree centrality, closeness centrality, betweenness centrality, and eigenvector centrality [34]. Here weights are assigned to these centrality measures as per their significance in biological networks for most influential node identification. Degree centrality is assigned a weight of 0.6, Betweenness centrality is assigned with a weight value of 0.3 and closeness centrality is allocated a weight of 0.2, and eigenvector centrality is assigned 0.1 weights. These weights are assigned arbitrarily so as to make a total weight of node is 1. This weighted network is shown in Fig. 5b.

Overall analysis and weighted analysis performed on the network constructed considering Pearson correlation criteria shown in Fig. 5. In the network, nodes highlighted in red color indicate nodes satisfying all the four centrality measures so they are inferred as the most influential nodes in the disease network.

We have used well known statistical software ‘R’ for survival analysis, gene co-expression network construction, and analysis from https://cran.r-project.org/web/package to identify genetic biomarkers associated with GBM.

## Results and discussion

Gene co-expression networks are constructed from survival outcome associated genes of DNA methylation dataset by considering three correlation measures which are Pearson, Spearman, and Kendall separately. The network constructed using the Pearson correlation measure is found stable and considered for further analysis. From these networks, different centrality measures like degree, betweenness, closeness, and eigenvector are applied to this constructed network to analyze and identify the most important nodes (genes). Among the top 20 nodes identified after applying each centrality measure independently, a final network is constructed in R as shown in Fig. 5. Using visualization methods a network is constructed to significantly represent important genes satisfying all the centrality criteria. Most influential genes are highlighted in the red color node; those can be predicted as the most influential genes with respect to the faster progression of the GBM as shown in Fig. 5a. A weighted analysis is carried out to verify the importance of the centrality measure in the progression of the disease [35]. An arbitrarily weights are assigned to each centrality measure and accordingly, the network is constructed using color code to highlight the most influential node in red color shown in Fig. 5b.

For each gene co-expression network, top 20 nodes are identified satisfying each centrality criteria separately and then the nodes which are found common that is satisfying all the centrality measures are extracted. In these ways, nodes representing genes SEMA3B, APS, SLC44A2, MARK2, PITPNM2, SFRP1, PRLH, DIP2C, CTSZ, and KRTAP4.2 are found to be a most influential node (highlighted in red color) and presented as a genetic biomarker associated with faster progression of the GBM. Developed code is uploaded in the GitHub repository with the link https://github.com/redekarseema2021/imp-genes.

**Fig. 6:**
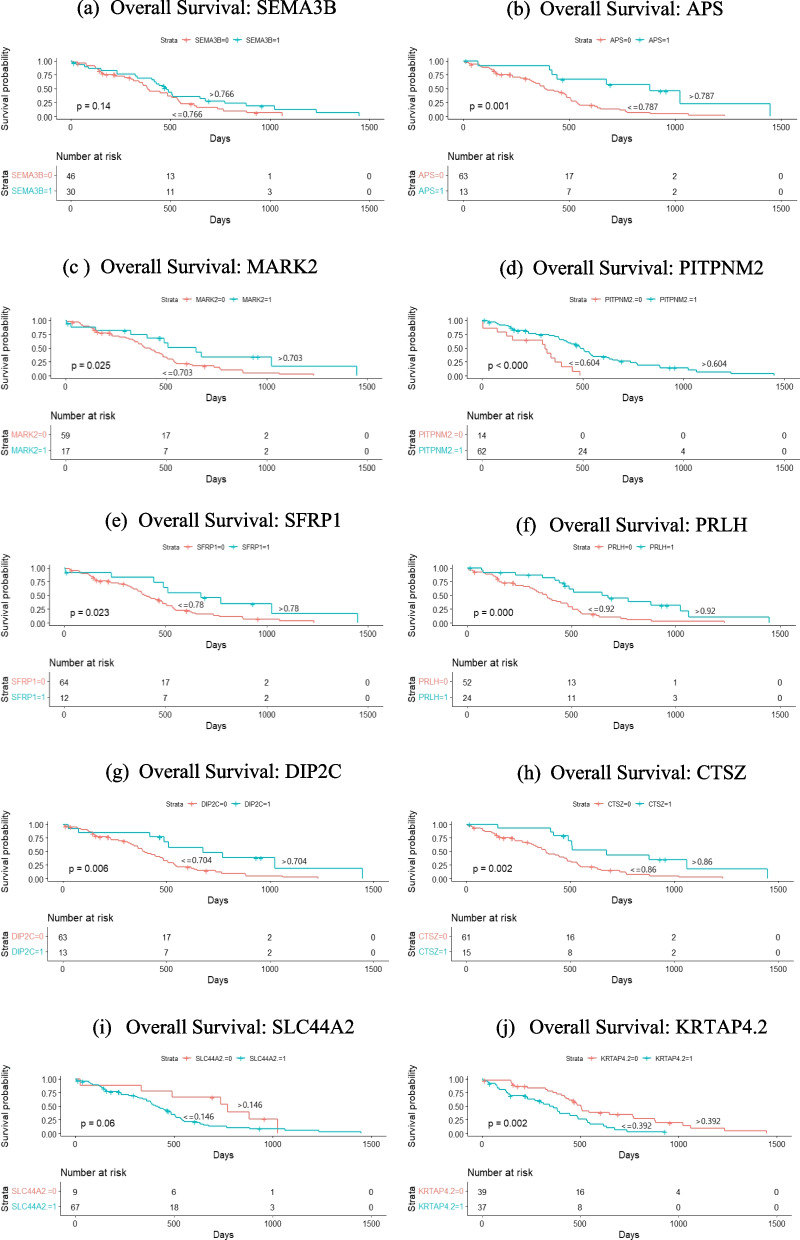
Kaplan–Meier curves indicating overall survival comparison of key genes at their threshold values. **a** Overall survival: SEMA3B. **b** Overall survival: APS. **c** Overall survival: MARK2. **d** Overall survival: PITPNM2. **e** Overall survival: SFRP1. **f** Overall survival: PRLH. **g** Overall survival: DIP2C. **h** Overall survival: CTSZ. **i** Overall survival: SLC44A2. **j** Overall survival: KRTAP4.2

These 10 influential genes are further analysis to find their effect on the progression of the disease by performing univariate analysis in R. The result is documented in the Table 2. Hazard ratio (HR) of the two genes SLC44A2 and KRTAP4.2 is greater than 1 which indicates that these genes are positively associated with the progression that means higher expression values of these genes are linked with the faster progression (earlier death). Whereas lower expression values of the genes named SEMA3B, APS, MARK2, PITPNM2, SFRP1, PRLH, DIP2C, and CTSZ are associated with the faster progression of the GBM as their hazard ratio is less than 1. Kaplan–Meier curve also shows the similar pattern and there is significant difference is observed between higher and lower threshold as shown in Fig. 6. These threshold values are computed by constructing Classification and Regression Tree (CART) [36].

**Table 2:** Univariate analysis of the key genes associated with GBM progression

Probe ID	Gene	HR [95% LCL, 95%UCL]	*P* value
cg08097657	SEMA3B	0.017 [0.001, 0.278]	0.004
cg16880396	APS	0.038 [0.004, 0.350]	0.003
cg21663431	SLC44A2	6.867 [1.759, 26.81]	0.005
cg17998964	MARK2	0.113 [0.024, 0.527]	0.005
cg08176694	PITPNM2	0.049 [0.007, 0.340]	0.002
cg06166767	SFRP1	0.117 [0.028, 0.488]	0.003
cg26427308	PRLH	0.001 [0.000, 0.177]	0.007
cg00025991	DIP2C	0.010 [0.000, 0.233]	0.004
cg23679724	CTSZ	0.007 [0.000, 0.203]	0.004
cg23205633	KRTAP4.2	7.057 [1.811, 27.51]	0.005

### Survival analysis of key genes

Kaplan–Meier survival analysis indicating overall survival comparison of the key genes is conducted. K-M curves of genes SLC44A2 and KRTAP4.2 shows that higher expression values of these genes are linked with faster progression of GBM as shown in Fig. 6i, j and lower expression values of genes SEMA3B, APS, MARK2, PITPNM2, SFRP1, PRLH, DIP2C, and CTSZ are associated with faster progression shown in Fig. 6a–h. Their cut-off values obtained through CART analysis is also recorded in K-M plots.

## Conclusion

A methodology is proposed to construct the gene co-expression network using Pearson correlation measure and then to analyze it using topological properties of the network, based on centrality measures. We have used the graph theory approach to identify most influential nodes representing key genes associated with the progression of the Glioblastoma multiforme. This analysis is advantageous to find genetic biomarkers associated with the progression of GBM which in turns linked with earlier death of GBM patient. These identified potential genes SEMA3B, APS, SLC44A2, MARK2, PITPNM2, SFRP1, PRLH, DIP2C, CTSZ, and KRTAP4.2 are analyzed to find their effect on the progression of the GBM. The survival analysis of these potential biomarkers is carried out to realize its correlation with the progression of the disease. Higher expression values of SLC44A2 and KRTAP4.2 are linked with the progression and lower expression values of SEMA3B, APS, MARK2, PITPNM2, SFRP1, PRLH, DIP2C, and CTSZ genes are linked with the progression. The expression levels of these identified genes can be accelerated or decelerated to control the progression of the disease as well as to tailor appropriate cancer therapy.

## Data Availability

Datasets obtained for this study is freely available at https://portal.gdc.cancer.gov/.

## Abbreviations

GBM: Glioblastoma multiforme
TCGA: The Cancer Genome Atlas
DNA: Deoxyribonucleic acid
GDC: Genomic Data Commons
OS: Overall survival
